# Tumor-on-a-chip model for advancement of anti-cancer nano drug delivery system

**DOI:** 10.1186/s12951-022-01552-0

**Published:** 2022-07-20

**Authors:** Chutong Tian, Shunzhe Zheng, Xinying Liu, Ken-ichiro Kamei

**Affiliations:** 1 Department of Pharmaceutics, Wuya College of Innovation, Shenyang Pharmaceutical University, Shenyang, 110016 Liaoning People’s Republic of China; 2 Chinese People’s Liberation Army 210 Hospital, 116021 Dalian, People’s Republic of China; 3 Institute for Integrated Cell-Material Sciences (WPI-iCeMS), Kyoto University, Yoshida-Ushinomiya-cho, Sakyo-ku, 606-8501 Kyoto, Japan

**Keywords:** Tumor-on-a-Chip, Microfluidic device, Nanomedicines, Drug delivery process, Preclinical prediction

## Abstract

Despite explosive growth in the development of nano-drug delivery systems (NDDS) targeting tumors in the last few decades, clinical translation rates are low owing to the lack of efficient models for evaluating and predicting responses. Microfluidics-based tumor-on-a-chip (TOC) systems provide a promising approach to address these challenges. The integrated engineered platforms can recapitulate complex in vivo tumor features at a microscale level, such as the tumor microenvironment, three-dimensional tissue structure, and dynamic culture conditions, thus improving the correlation between results derived from preclinical and clinical trials in evaluating anticancer nanomedicines. The specific focus of this review is to describe recent advances in TOCs for the evaluation of nanomedicine, categorized into six sections based on the drug delivery process: circulation behavior after infusion, endothelial and matrix barriers, tumor uptake, therapeutic efficacy, safety, and resistance. We also discuss current issues and future directions for an end-use perspective of TOCs.

## Introduction

Cancer is the second leading cause of death worldwide, with the global cancer burden aggravated year by year. According to the latest data published in GLOBOCAN by the IARC, there would be 50% increase in the cancer burden and estimated 30 million cancer cases by 2040 [1]. Despite extensive efforts to improve anti-cancer treatments as well as cancer prevention and screening, the global cancer statue remains a major issue worldwide.

The usage of the anti-cancer drugs is always limited by the drug solubility or stability, poor pharmacokinetics behavior, lack of tumor specificity and severe side-effects. In recent years, with the development of nanotechnology, a large amount of nano anti-cancer therapeutics flourished [2–5]. These studies focused on delivering the anti-cancer agents to tumor sites specifically without or decreasing side effects. Basic nano-drug delivery system (NDDS) including polymer nanoparticles (NPs) [6, 7], liposomes [8, 9], micelles [7] and inorganic NPs [10, 11] to treat cancer offer benefits over conventional anti-cancer drug delivery systems such as improved drug solubility or stability without addition of toxic surfactants, increased tumor distribution due to enhanced permeability and retention effect (EPR effect) and so on. Tumor cell heterogeneity provides a basis for the design of active targeted and selectively released NDDS [12–15]. Tumor specificity and selective release, preventing off-target effects, are key properties of these systems. In addition, with the deep study in physiology, the artificial or biomimetic materials are emerging, such as protein carriers [16], cell membrane-coated NPs [17], exosomes [18], viruses [19] and bacteria-like NPs [20]. These bio-derived nanostructures are biocompatible or naturally tumor-targeted, improving safety, plasma circulation time, and tumor specificity.

In addition to targeting tumor cells directly, recent research has focused on the treatment of the tumor microenvironment (TME) as a whole [21, 22]. The components of the TME, including various cell types, surrounding stroma, blood vessels and lymphatic vessels, and cytokines [23], provide a good material basis for the proliferation and metastasis of tumor cells and serve as barriers or chances for anti-cancer nanomedicines [24]. For example, the rigid tumor extracellular matrix (ECM) impedes drug penetration. However, in some cases, it can also be the target of nanomedicines. Similarly, new-born vessels in tumors can promote aggression and metastasis but also can play important roles in EPR effect-based nanomedicine delivery. Overall, despite promising results of preclinical research, most of them fail to achieve clinical success. One of the biggest challenges is the models used for evaluation.

Animal models are important for in vivo preclinical evaluation owing to the ability to evaluate total responses of anti-cancer medicines. Commonly used subcutaneously implanted models always lack features of the native tissue-specific microenvironment. In response, in vivo orthotopic cancer models showed better fidelity. However, critical issues with these animal models limit the evaluation of the efficiency and toxicity of drugs, including interspecific differences, leading to a poor predictive ability for therapeutic responses in human clinical trials [25–27]. Furthermore, animal models are ineffective for analyses of specific physiological or molecular mechanisms owing to the complexity of animal physiology.

In vitro cell culture models have been used to address issues associated with animal models (Table 1). Conventional 2D tumor cells have been used to indict the cancer cytotoxicity of drugs for a long time. Although conventional 2D cell culture platforms can be performed in an inexpensive and high-throughput fashion, they often have limited predictive value for the drug response [28, 29], because they cannot recapitulate the in vivo TME, which may mislead or exaggerate the results regarding drug sensitivity to tumor cells. To tackle these issues, in vitro evaluation methods based on 3D tumor spheroids have been developed, in which tumor cells with or without other caner-associated cells are cultured in 3D ECM gel [30]. 3D spheroid models can reproduce some TME cues, such as cell-cell/ECM interactions and oxygen/nutrient gradients [31]. Organoid technology with the use of patient specimens has emerged in recent years and is considered to recapitulate the pathophysiological TME in vitro for applications in drug evaluation as well as personalized medicine. Although both 3D spheroid and organoid models show significant improvements over 2D cell culture platforms, various issues still need to be addressed, such as physical TME cues. For example, static culture conditions are generally used for spheroids/organoids, but cannot reproduce shear stresses/hydroid pressures and inter-tissue interactions via blood/interstitial flow and vascular perfusion [30, 32–34]. Therefore, even models using patient specimens cannot re-create the inherent functions of in situ organs (for example, peristalsis in intestine), which may influence drug delivery behavior [27, 35]. Hence, there is an urgent need to develop an effective evaluation model able to recapitulate the complicated features of the TME, and realize the accurate selection of nano-therapeutic agents for patients.

**Table 1:** Advantages and disadvantages of different in vitro tumor culture models

Models	Advantages	Disadvantages
2D cell-culture	Cheap, High-throughput Easy to culture	Only tumor cell lines No TME Morphology and phenotype changes
3D spheroids	Cell-cell and cell-ECM interaction Physiological morphology	Batch-to batch variation Lack of vascularization and shear stress
Organoids	Patient-specific cell High heterogeneity	Expensive and difficult to culture Lack of vascularization and shear stress Lack of inherent functions of in situ organs

**Scheme 1:**
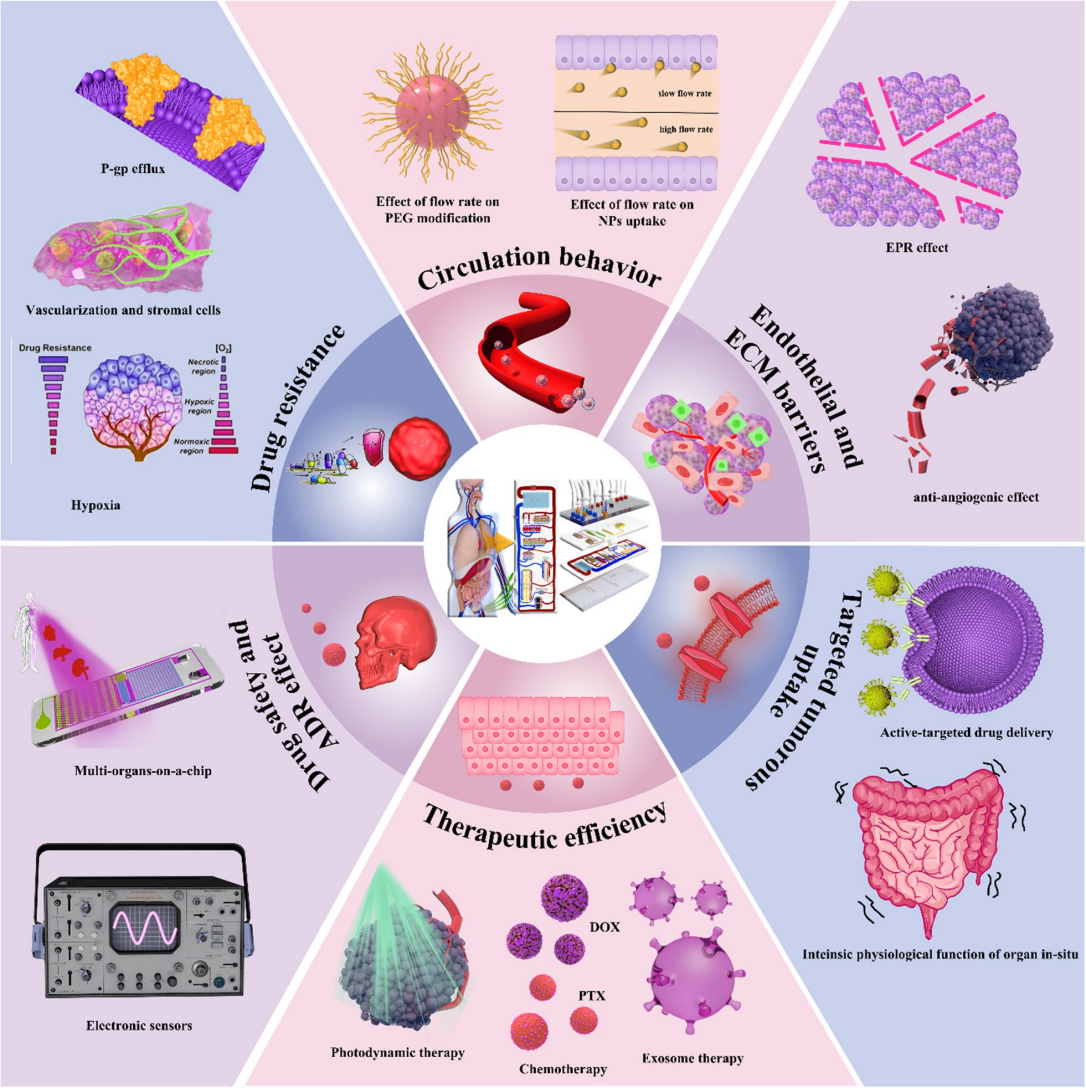
Application of TOC platforms in every important step of the NDDS delivery, including circulation behavior after infusion, endothelial and ECM barriers, tumorous uptake, therapeutic efficiency as well as evaluation of drug safety and resistance. Every step can be influenced by varied aspects

Recently, organ-on-a-chip (OOC) has offered enormous potential to bridge the gap between in vitro evaluation models and in vivo pathophysiological complexity [36, 37]. Particularly, tumor-on-a-chip (TOC) platforms can mimic structures, functions and biological process in living tumors, enabling efficient evaluations of advanced anti-cancer NDDS [27, 38]. Here, we review on-going research directed towards the anticancer NDDS response on TOCs, including the aspects of circulation behavior after infusion, endothelial and ECM barriers, tumor uptake, therapeutic efficiency as well as evaluation of drug safety and resistance (Scheme 1).

## Tumor-on-a-Chip

**Fig. 1:**
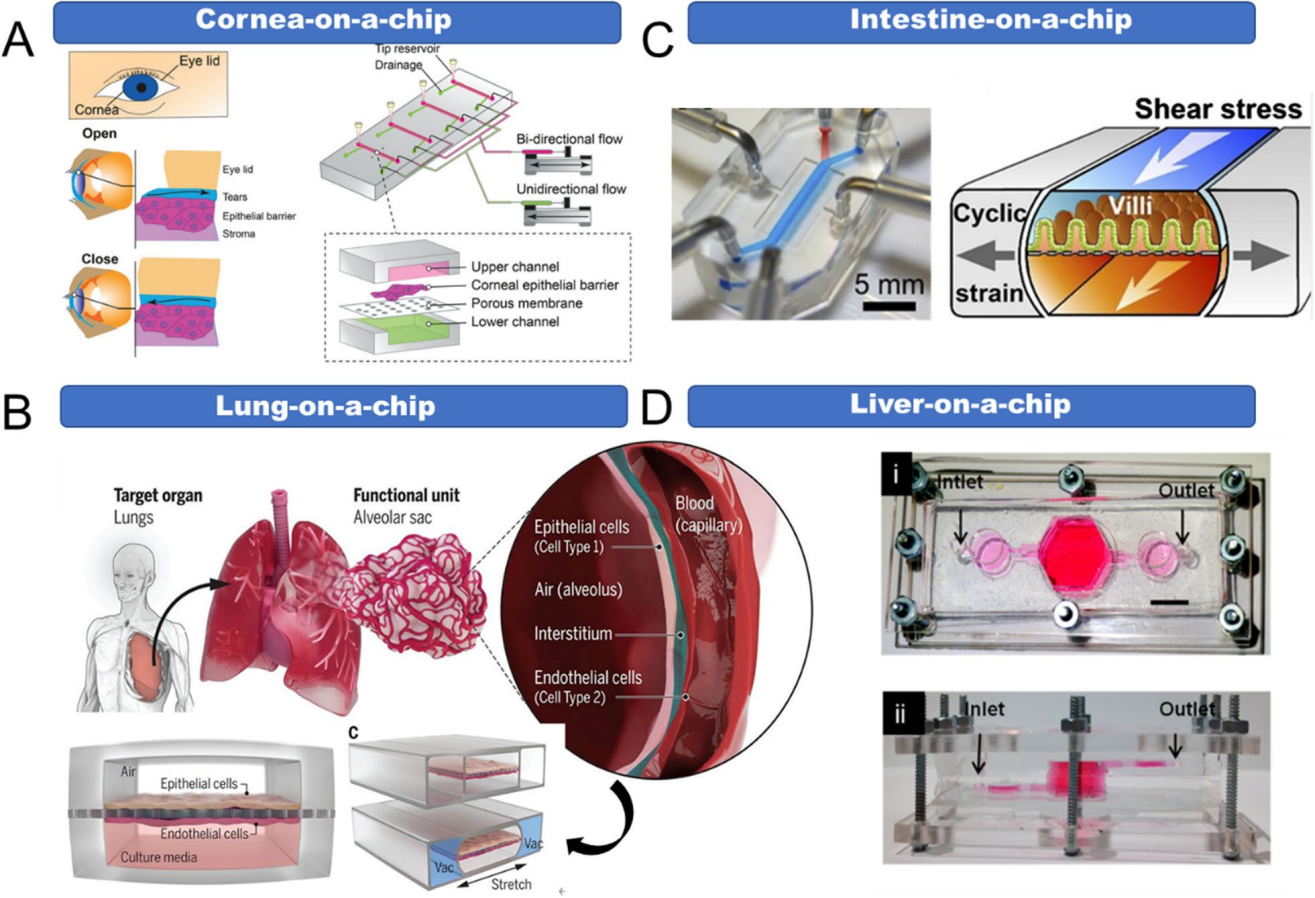
Reverse engineering in the design of the organ-on-a-chip. **A** Cornea-on-a-chip reflecting the blinking process. Reproduced with permission [41], Copyright 2020, RSC publications. **B** Lung-on-a-chip reflecting the breathing process. Reproduced with permission [40], Copyright 2010, Science publications. **C** Intestine-on-a-chip reflecting peristalsis. Reproduced with permission [42], Copyright 2014, Nature publishing group. **D** Liver-on-a-chip reflecting metabolic function. Reproduced with permission [43], Copyright 2016, IOP Publishing

OOC platforms are cell culture devices based on microfluidic technology. They integrate tissue cells with physiologically relevant microenvironments, simulating the physiological functions of human organs in vitro [39]. It is difficult to fully reconstruct living systems in vitro; hence organ chips employ “reverse engineering”, which aims to extract the specific functions from target organs (Fig. 1). For example, this approach was used to construct the classical lung-on-a-chip [40]. The chip was composed of two layers separated by a porous membrane; the upper layer cultures pulmonary epithelial cells and the lower layer cultures vascular endothelial cells. There are vacuum side channels on both sides of the chip, which expand and contract by circulating suction, thereby driving the contraction of cells on the membrane to mimic the breathing function of the living lung (Fig. 1B).

Since firstly raised by Prof. Shuler et al., OOC platforms have been developed rapidly in recent years. A variety of organ chips such as liver [43–45], lung [40], heart [46, 47], kidney [48], intestine [49] and multi-organ-on-a-chips [50, 51] have been fabricated. They are widely used for disease research, new drug development, toxicity evaluation, and so on. As cancer has become one of the most serious causes of death this century, the use of TOCs to model anticancer drug responses has attracted the attention of many researchers [52–64]. TOCs are OOCs in which healthy tissue cells are replaced with tumor cells. They could demonstrate the interactions of tumor cells with related cells and changes in the ECM in the 3D TME, reflecting the dynamic processes involved in tumor development. Furthermore, TOCs could re-create in vivo cellular systems in a simple and inexpensive way, allowing for high-throughput and multiplexed drug screening at the organ and systemic levels [65]. These platforms are more accurate and sensitive responses to therapeutic effects and are therefore conducive to the development of NDDS. Overall, TOCs are expected to generate significant advances in drug screening, especially in smart nanomedicines designed to target the heterogeneous TME.

## Tumor-on-a-chip platforms for preclinical evaluations of nano-drug delivery systems

In this section, we introduce the application of TOC platforms in every important step of the NDDS, including the circulation behavior after infusion, endothelial and ECM barriers, targeted tumorous uptake as well as evaluation of therapeutic efficiency.

### Circulation behavior of NDDS

NDDS are usually administrated by intravenous infusion, which exposes them to the systemic circulation and travel throughout the body [66]. In this process, the NDDS would interact with the dynamic bloodstream, which may impact in vivo fates substantially. However, it is very difficult for conventional in vitro methods to recapitulate the fluid flow characteristics of the bloodstream [67]. Additionally, established in vitro assays often incubate the NDDS with cancer cells directly, ignoring the NDDS stability in circulation and interactions with endothelial cells [68, 69]. Microfluidic TOC platforms with endothelial cells allow the characterization of the fluid flow rate and shear stress of NDDS in the dynamic circulatory bloodstream. When subjected to flow, endothelial cells are metabolically active and shear-responsive with more intact tight junctions [70]. These phenotypic properties could affect the trans-endothelial ability of NDDS. For example, Chen et al. employed cy5-taged gold nanoparticles to determine the effect of flow rate on endothelial uptake. The results suggested the fluid flow rate is inversely related to the nanoparticles uptake [71].

In addition to NDDS endothelial cell interactions, the stability of NDDS in the blood stream is also an important factor. The blood shear stress and flow rate may lead to NP disassembly before reaching tumor cells. 3D TOC platforms in combination with advanced imaging techniques provide an opportunity to track the conformation of supramolecular micelle nanocarriers continuously during their delivery in vessels and the TME. These TOC platforms provide a new approach to evaluate nanocarrier candidates efficiently in vitro [72]. Besides, to get a longer half-life, NP surfaces are always modified e.g. by polyethylene glycol (PEG) [73]. Some target ligands may also be decorated into PEG [74]. TOC platforms are able to illustrate the effect of the vascular flow on surface modified NPs. Maria. et al. reported a microvascular network device (MN) that mimics the complex vasculature and fluid flow conditions in vivo [75]. The stability of the PEGylated or folic acid (FA)-headed camptothecin NPs was analyzed using the MN, revealing that the blood flow would induce PEG detachment from physically adsorbed PEGylated NPs, while endothelial cells have no significant effect. They also detected significant changes in zeta potential after exposure to flow and flow-induced contact with the wall with endothelia cells. Surface modification is a common strategy used in NDDS; for example, the FDA approved nanomedicine, Doxil, in which PEG coating, improves its pharmacokinetic (PK) behavior [76]. However, traditional 2D cell-culture model is difficult to recapitulate the dynamic circulation. It is also difficult to monitor the status of NDDS in real time with animal models. The TOC vascular system provides a chance to make this grey process clear.

### Endothelial and ECM barriers

**Fig. 2:**
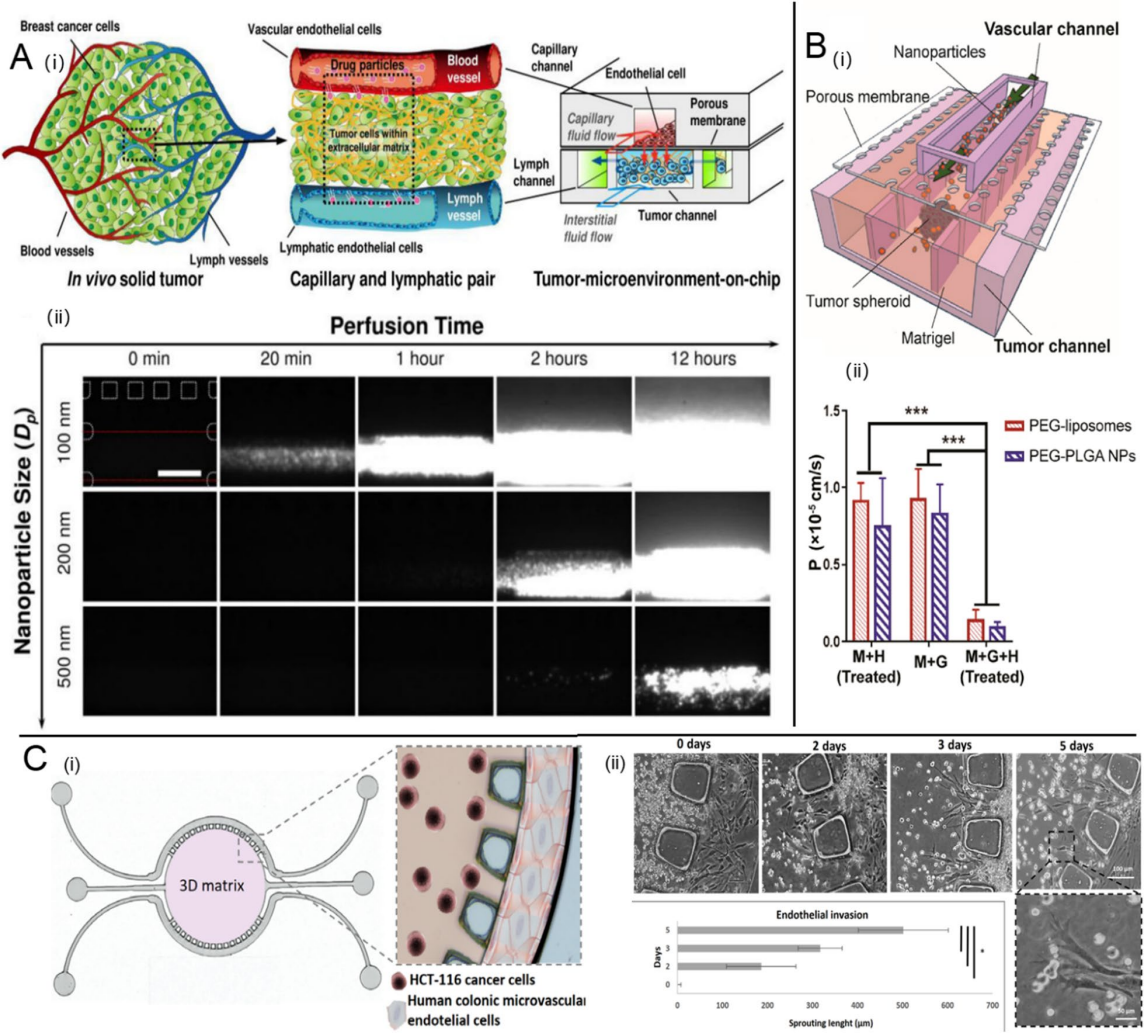
Effect of endothelial and ECM barriers on nano-drug delivery. **A** “Tumor-microenvironment-on-chip” (T-MOC) for NP transport assessment. (i) schematic diagram of design principles, (ii) characterization of the effects of NP size on extravasation and interstitial diffusion using T-MOC. Reproduced with permission [77], Copyright 2014, Elsevier publications. **B** Tumor-vasculature-on-a-chip (TVOC) model to recapitulate the EPR effect, (i) schematic diagram of design principles, (ii) Permeability coefficients for NPs in TVOC. Reproduced with permission [53], Copyright 2018, ACS publications. **C** 3D colorectal tumor-on-a-chip to assess the nano-drug delivery efficiency, (i) schematic diagram of design principles, (ii) formation of endothelial sprouts in the microfluidic device. Reproduced with permission [54], Copyright 2019, AAAS publications

After NPs reach tumor tissues, extravasation from the vessels and penetration through the dense extracellular matrix (ECM) are necessary before they are taken up by tumor cells. TOC platforms are useful for understanding how NDDS overcome these penetration barriers. Many studies have focused on the 3D tumor microarchitecture and dense ECM barriers. Huang et al. reported a 3D multicellular spheroid-on-a-chip system to model hydromechanical conditions that closely match the TME [52]. They showed that both the surface charge of NPs and the protein corona surrounding NPs affect penetration resistance. Further, vessel components of tumor tissues were added to the improved tumor chips to better mimic the in vivo TME. For instance, Kwak et al. created a “tumor-microenvironment-on-chip” (T-MOC) to recapitulate several key features of the TME during the NP transport process, such as tumor lymphatic and blood vessels, elevated tumor interstitial fluid pressure (IFP) and dense ECM (Fig. 2 A) [77]. The system was used to investigate the diffusive transport of transferrin (Tf) or PEG coated gold NPs. The NP penetration efficiency into complex 3D T-MOC was directly affected by the NP diameter. Due to aggressive tumor growth, tumor tissues always show leaky vasculature and defective lymphatic drainage, promoting NP accumulation and retention at the tumor site, also known as the EPR effect. The EPR effect is one of the most widely used principles in passive targeting NP design [78, 79]. An in vitro model that could recapitulate this special phenomenon would be promising for the design of effective NPs for tumor drug delivery. Hence, Wang et al. designed a tumor-vasculature-on-a-chip (TVOC) model involving endothelial barriers, rigid ECM barriers, and a 3D tumor spheroid structure (Fig. 2B) [53]. Tumor necrosis factor-α (TNF-α), the primary factor that induces endothelial barrier dysfunction, was added to the system to simulate the impaired tumor vasculature related to the EPR effect. PEGylated liposomes and poly (lactide-co-glycolic acid) (PLGA) NPs were prepared by a one-step microfluidic method and fluorescently labeled for imaging. They were then added to the top channel of the TVOC at a certain flow rate. The effects of sole TNF-α-treated endothelia barrier (M + H Treated) or ECM barrier (M + G) or the combination of barriers (M + G + H Treated) on NP permeability were investigated in detail by quantifying NPs in the bottom channel (Fig. 2B(ii)). Either factor alone had a much weaker effect on NP transport than that of the combination of both barriers. Additionally, the permeability coefficients (P-value) for PEGylated liposomes and PLGA NPs obtained in the TVOC with both endothelial and ECM barriers was only slightly higher than that obtained from animal models, verifying the efficacy of the TVOC to recapitulate the EPR effect in vitro and the ability to assess NP transport. In another recent study, Carvalho et al. developed a 3D colorectal TOC to assess the delivery efficiency of gemcitabine (GEM)–loaded NPs (Fig. 2 C) [54]. The TOC platform was composed of a circular ECM–like hydrogel channel with colorectal tumor cells, and two side channels implanted with endothelial cells. The GEM-loaded NPs were added to the vascular side channel, resulting in an antitumor effect dependent on the microfluidic-based dynamic controllable diffusive transport from the vascular compartment to the central tumor channel. In addition, vascular endothelial growth factor (VEGF) loaded in the hydrogel channel promotes microvasculature growth within the ECM channel as a gatekeeper, representing a key advance over previous work.

**Fig. 3:**
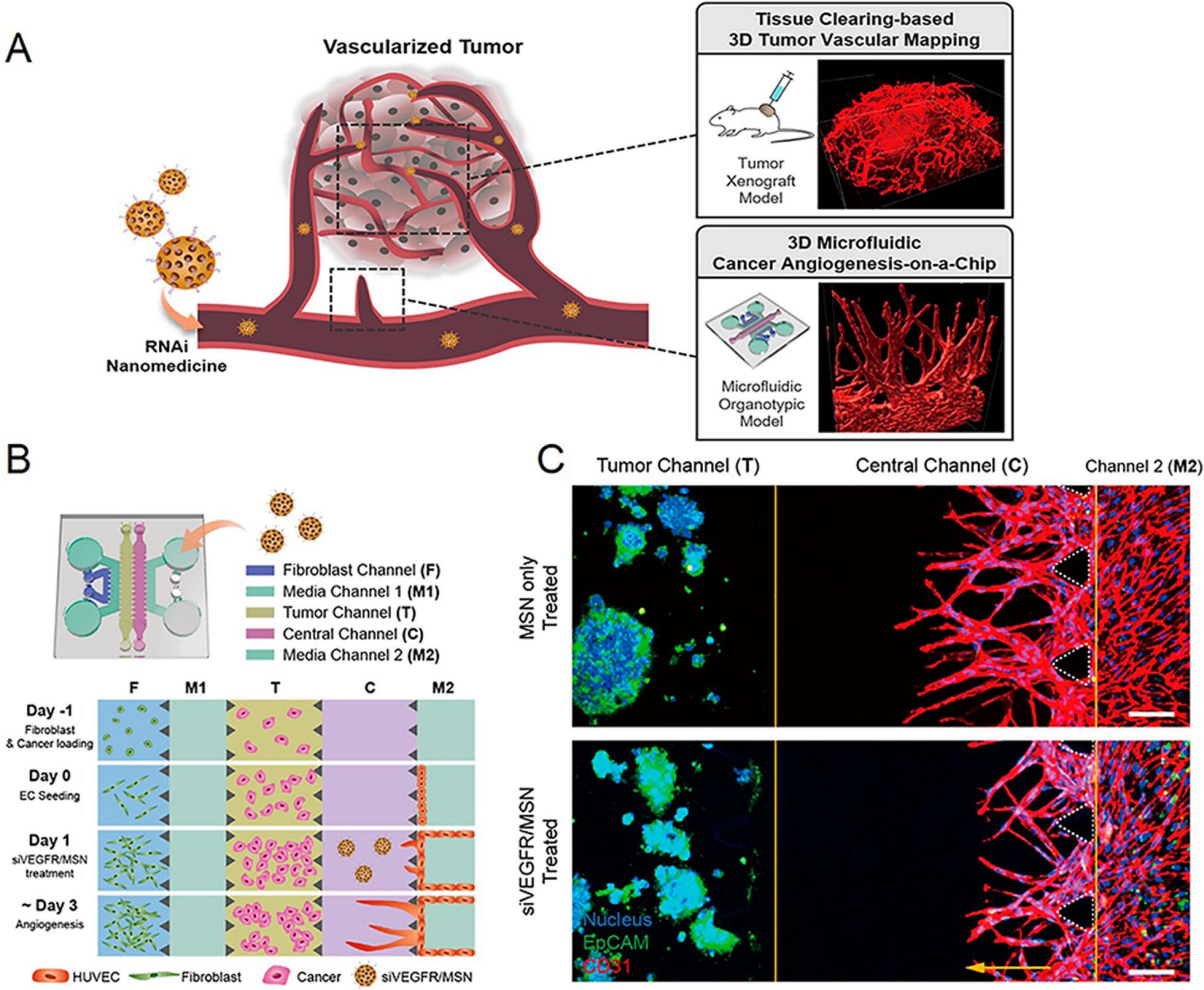
RNAi-based nanomedicine evaluation using 3D microfluidic cancer angiogenesis-on-a-chip, **A** Schematic diagram of design principles. **B** Schematic overview of the chip design for cancer angiogenesis assay. **C** Angiogenesis assay with or without siVEGFR/MSN treatment. Reproduced with permission [55], Copyright 2021, ACS publications

In addition to the barrier effects, the tumor microvasculature also acts as a nutrient transport network [80]. Anti-angiogenic drugs are widely used in clinic to destroy the new-born tumor microvasculature and cut off the nutrient/oxygen supplies, thus killing tumor cells [81, 82]. However, 2D in vitro models are not optimized for evaluating anti-cancer angiogenesis owing to the lack of the vessel-tumor interaction. 3D vascularized TOCs provide opportunities for the assessment of anti-angiogenic effects in vitro [83–85]. Lee et al. developed a microfluidic cancer angiogenesis-on-a-chip to evaluate anti-angiogenic siVEGF/VEGFR (small interfering RNA of vascular endothelial growth factor or its receptor)-loaded mesoporous silica nanoparticle (MSN) (Fig. 3) [55]. This chip includes a fibroblast channel to induce VEGF signaling, a M1 channel for the administration of siVEGF-loaded MSN, a tumor channel, a central channel for the administration of siVEGFR-loaded MSN and a M2 channel cultured with endothelial cells. In a comparison of the anti-angiogenic effect of siVEGF and siVEGFR-loaded MSN, the chip could screen out highly responsive siVEGFR MSN. The sprouting phenotypes in different cancer types were also clearly visualized and quantified using this model. HepG2 showed most sensitive response to the siVEGFR MSN, while SW620 or A549 showed weak or negative responses. Moreover, the validity of the siVEGFR MSN was also observed in an animal model, further verifying that the system could be a powerful platform for developing anti-angiogenic nanomedicines.

### Targeted tumor uptake

**Fig. 4:**
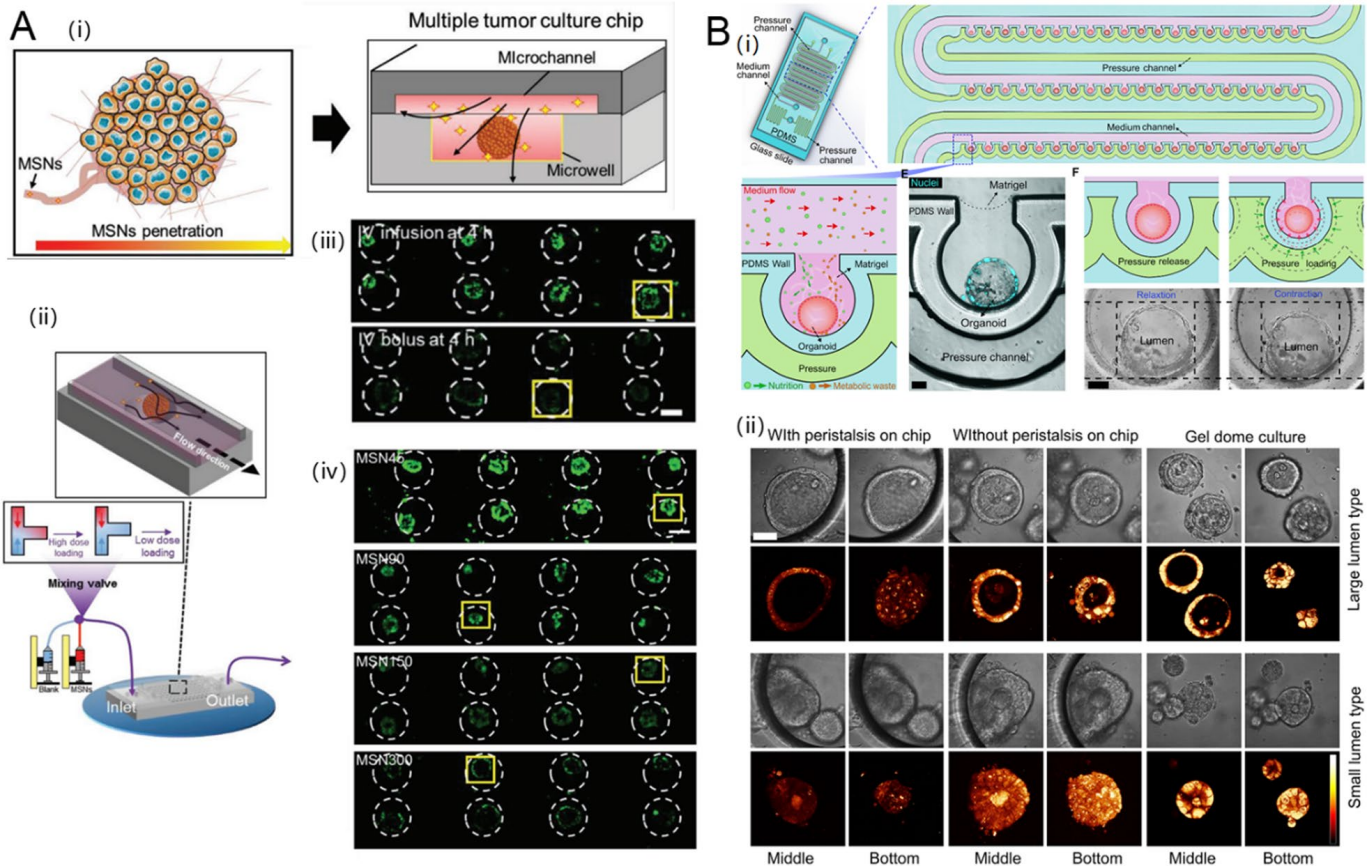
Targeted tumorous uptake of NDDS in TOCs. **A** Multiple tumor culture chip (MTC-chip) to assess the cellular uptake of MSNs, (i) schematic diagram of design principles, (ii) comparison of different routes of MSNs administration on the chip with three-way mixing valve inlet, (iii) characterization the effect of administration routes on MSN penetration, (iv) characterization the effect of MSN particle size on MSN penetration. Reproduced with permission [56], Copyright 2019, John Wiley and Sons. **B** Peristaltic human colon tumor organoids on the microfluidic chip to model the cellular uptake of micelles, (i) schematic diagram of design principles, (ii) micelle uptake in organoids cultured by different methods. Reproduced with permission [57], Copyright 2021, IOP Publishing

Many studies have demonstrated that NPs are beneficial with respect to cell uptake efficiency. However, the results of in vitro and in vivo studies differ substantially, which limits the clinical translation of NPs [86–89]. Aforementioned, this is the largely due to the limitations of current in vitro evaluation methods based on 2D cell-culture and tumor spheroids, which lack shear stress, interstitial fluid pressure, and 3D tumor tissue structures [90–92]. TOC platforms can account for 3D tumor scaffolds and fluidic shear stress. For example, Zhuang et al. fabricated a multiple tumor-culture chip (MTC-chip), which integrated 3D tumor spheroids, ECM, and dynamic administration into one system to assess the cellular uptake and penetration depth of MSNs (Fig. 4A) [56]. The dynamic and adjustable drug import of the MTC enables analyses of the effect of different administration routes on the tumor penetration of MSNs. They compared the tumor penetration of MSNs in TOC with continuous administration (e.g., IV infusion) and transient administration (e.g., IV bolus). There was one drug-containing inlet pump and a blank medium-containing inlet pump (Fig. 4 A(ii)). By adjusting different flow rates of these two pumps, the drug import concentration could be varied to reflect two different administration routes. Tumor accumulation after transient administration (IV bolus) decreased rapidly and was only distributed at the edge of the spheroids, while continuous dynamic treatment with MSNs resulted in greater and deeper penetration. These results suggest that the most effective route for the administration of NPs, like MSNs, to achieve high tumor accumulation, is continuous, rather than transient. Furthermore, larger MSNs that are not taken up in 2D cell models or static conditions could diffuse into tumor cells in a lower efficiency in the MTC model. This result means that traditional 2D cell models may amplify the effect of particle size on cellular uptake, missing some promising NPs that may perform well in vivo. Unlike other tumors, it is essential to re-create peristalsis for in vitro digestive tract tumor models [93]. Fang et al. presented human colon tumor organoids on a microfluidic chip enabling the recreation of peristalsis (Fig. 4B) [57]. They implemented a pressure channel to surround tumor organoids containing a microwell array, providing peristalsis amplitude and rhythm. This peristaltic colon tumor organoid chip was used to investigate the cellular uptake of ellipticine-loaded micelles. Uptake was distinctly lower compared to that on the chip without peristalsis. These kinds of chips with physiological functions, such as peristalsis, may enable more acute evaluation on the effectiveness of nanomedicines for tumors.

Active-targeting NDDS have also been proved having great potency in cancer therapy [94, 95]. They largely depend on the specific tumor-targeting ligands decorated on surface of the NPs. Active-targeting NDDS could improve selective tumor accumulation through target-receptor effect [3, 96]. They always show highly efficient tumor cellular uptake on 2D cell models, while fail or inefficient (only 2%) in in vivo [97]. This discrepancy could be improved by TOC models. In the study of Wang et al. mentioned above, they also evaluated the uptake of folate receptor-targeted liposomes (FA-liposomes) and PEG-PLGA nanoparticles (FA-PLGA NPs) on 2D monolayer cells, 3D tumor spheres, and TVOC models [53]. On 2D monolayers and 3D tumor spheroids, the cellular uptake of targeted NPs was stronger than non-targeted NPs. However, cellular uptake of targeted and non-targeted agents did not differ significantly in the TVOC model, consistent with animal models, confirming the similarity between TVOC and in vivo models. The effectiveness of the TOC model compared with the 2D or 3D spheroid models may be attributed to the emphasis of hinderance between NPs and receptors on tumor cells caused by fluidic shear stress and biological barriers.

### Evaluation of the therapeutic efficiency of diverse NDDS

**Fig. 5:**
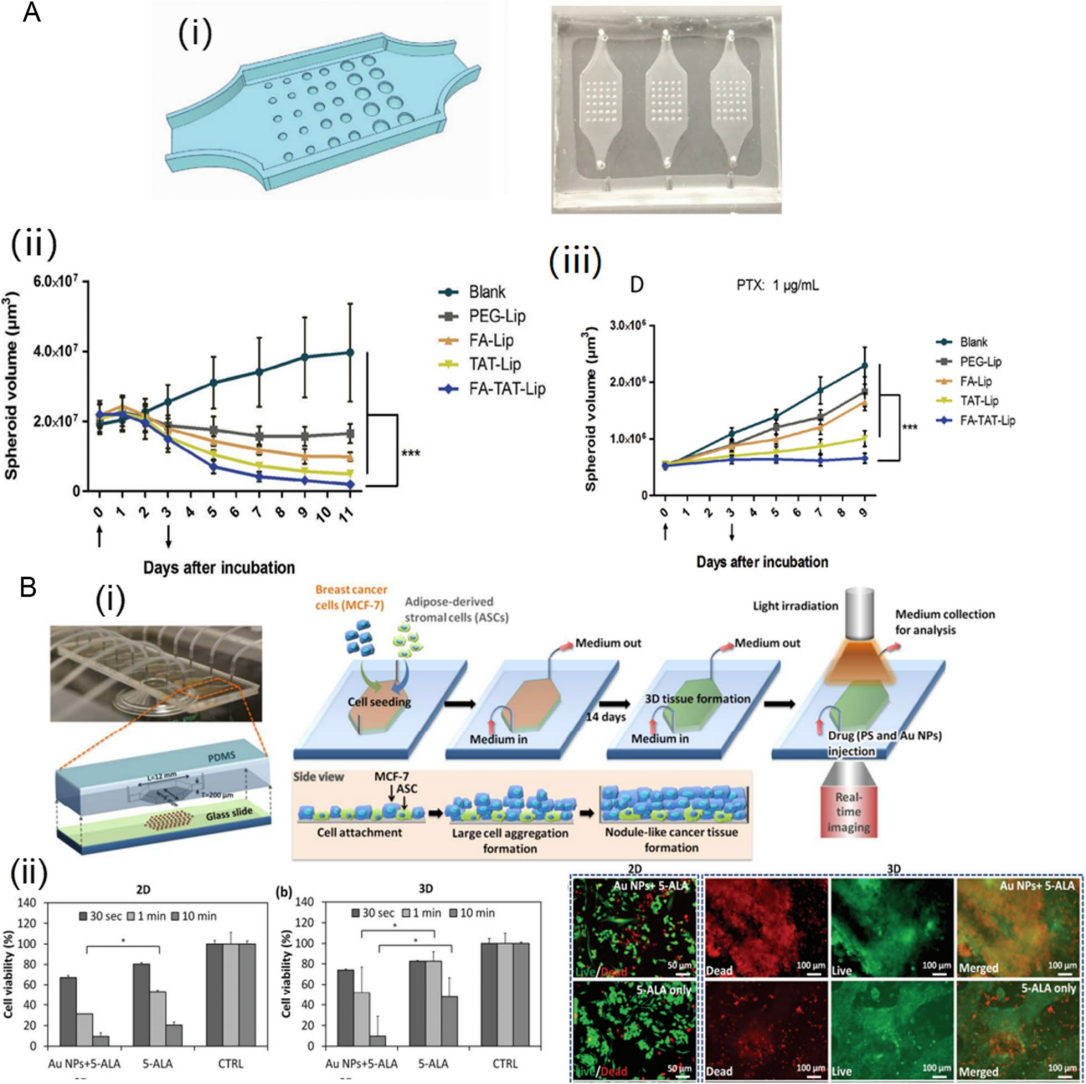
Evaluation of the therapeutic efficacy in TOCs. **A** Microfluidic TOC to assess the multifunctional liposome anticancer efficiency, (i) schematic illustration of the TOC device design and the TOC device after soft lithography, (ii) tumor suppressive effecta using tumor spheroid model, (iii) tumor suppression efficacy study using the TOC model. Reproduced with permission [58], Copyright 2019, John Wiley and Sons. **B** 3D microfluidic breast cancer-on-a-chip for determining the efficiency of PDT, (i) schematic illustration of the TOC device design, (ii) cell destruction was measured on both 2D monolayer culture and 3D microfluidic tissue culture 24 h after various PDT treatments. Reproduced with permission [59], Copyright 2015, RSC publications

Based on the detailed mechanisms underlying tumor progression, diverse NDDS have been developed to cure tumors, such as targeted chemotherapeutic NDDS, PDT-based NDDS, exosomes or NDDS eliciting anti-tumor immunity. The TME plays important roles in their antitumor effects. Microfluidic platforms may offer a faster and more economical alternative to 2D cell culture systems and in vivo animal models for evaluations of complicated NDDS. For example, Ren et al. designed a microfluidic TOC to assess the multifunctional liposome anticancer efficiency (Fig. 5 A) [58]. They included 30 hemispheric wells with three different radii to fabricate tumor spheroids of different sizes, allowing the screening of diverse NPs on the chip simultaneously. The antitumor effect of paclitaxel (PTX)-loaded PEGylated liposome (PEG-Lip) or targeted liposome modified by folic acid (FA-Lip), cell-penetrating peptide TAT (TAT-Lip) or both folic acid and TAT (FA-TAT-Lip) were evaluated on 2D monolayer, 3D spheroid, and TOC models, respectively. The effect of the same formulation on different models decreased in the following order: 2D monolayer > 3D spheroid > TOC. Specially, instead of reducing the sizes of the tumor spheroids as observed in the 3D spheroid model, there was only a limited tumor growth suppression effect of PTX-loaded liposomes showed on the TOC model. The phenomenon was consistent with the results obtained using the animal model. In addition, the effects of flow rate on the therapeutic efficacy were also investigated on the TOC model. An elevated flow rate resulted in a reduced antitumor effect for the dual-targeted PTX-loaded liposomes, which may be attributed to lower NP uptake with a higher interstitial flow rate.

In addition to screening chemotherapeutic NDDS, TOCs also be advantageous in evaluation effect of other therapeutic strategies, such as photodynamic therapy (PDT). The limited efficacy of the PDT has two main explanations: (i) poor light penetration to deep tissue; (ii) PDT resistance caused by tumor hypoxia. However, the 2D monolayer model could not recapitulate either these two conditions [98, 99]. Yang et al. established a 3D microfluidic breast cancer-on-a-chip to evaluate the effect of PDT using a photosensitizer and Au NPs (Fig. 5B) [59]. The 3D tumor tissue could be formed by the introduction of MCF-7 and adipose-derived stromal cells (ADSCs) and dynamic culture on the chip. The microfluidic chip can mimic the heterogeneous TME and the 3D structure of the tumor tissue, providing penetration depth and hypoxia conditions. Compared with 2D monolayer culture, breast cancer cells cultured in a 3D microfluidic model showed stronger resistance to PDT. The poor PDT response in the 3D model may be explained by (i) an oxygen deficiency associated with the high cell density in 3D cancer tissue and (ii) poor penetration of the photosensitizer due to dynamic infusion and the tissue depth. This study demonstrated that the microfluidic chip has good reliability and physiological accuracy for the prediction of PDT outcomes.

Exosomes can act as natural messengers between cells to deliver bioactive molecules, such as RNAs and proteins, and hence have attracted significant attention in recent research. To better illustrate the efficacy of exosome-based therapeutics, a complex and multi-cellar in vivo-like microenvironment should be developed. Jeong et al. constructed a microfluidic 3D lung cancer model that includes cell-cell communications between lung tumor cells and endothelial cells [100]. Using the 3D model, they demonstrated that miR-497-loaded exosomes have synergistic inhibitory effects on both endothelial cells tube formation and tumor cell migration. The study indicated that TOCs are also predictive tools for evaluating emerging biomimetic drug delivery systems, such as exosomes. Besides, the combination of chemotherapy with immunotherapy yields an improved anticancer effect with both the quick-killing effect of chemotherapy and the long-term effect of immunotherapy. With the expanding number of novel NDDS able to elicit combination therapeutic effects, there is increasing interest in the use of TOCs to interrogate the tumor immune microenvironment. For instance, Bijay et al. used a microfluidics-based 3D, compartmentalized breast cancer-on-a-chip (BCC) to study immune cell recruitment by the developed NDDS [101]. In their BCC, breast cancer cells were loaded in the bottom chamber and THP-1 cells (substitutes of macrophages in the in vitro culture) were circulated through the fluidic channel. The administration of hyaluronic acid (HA) NP conjugated with the chemotherapy drug gemcitabine (GEM) and the immunomodulatory drug imiquimod (IMQ) in the BCC resulted in greater number of THP-1 cells migration into the breast tissue chamber compared with that for the NPs conjugated with GEM alone, verifying the NDDS could harbor IMQ to promote the infiltration of immune cells in tumor tissues and supporting their clinical value in combination therapy.

### Drug safety and adverse effects

**Fig. 6:**
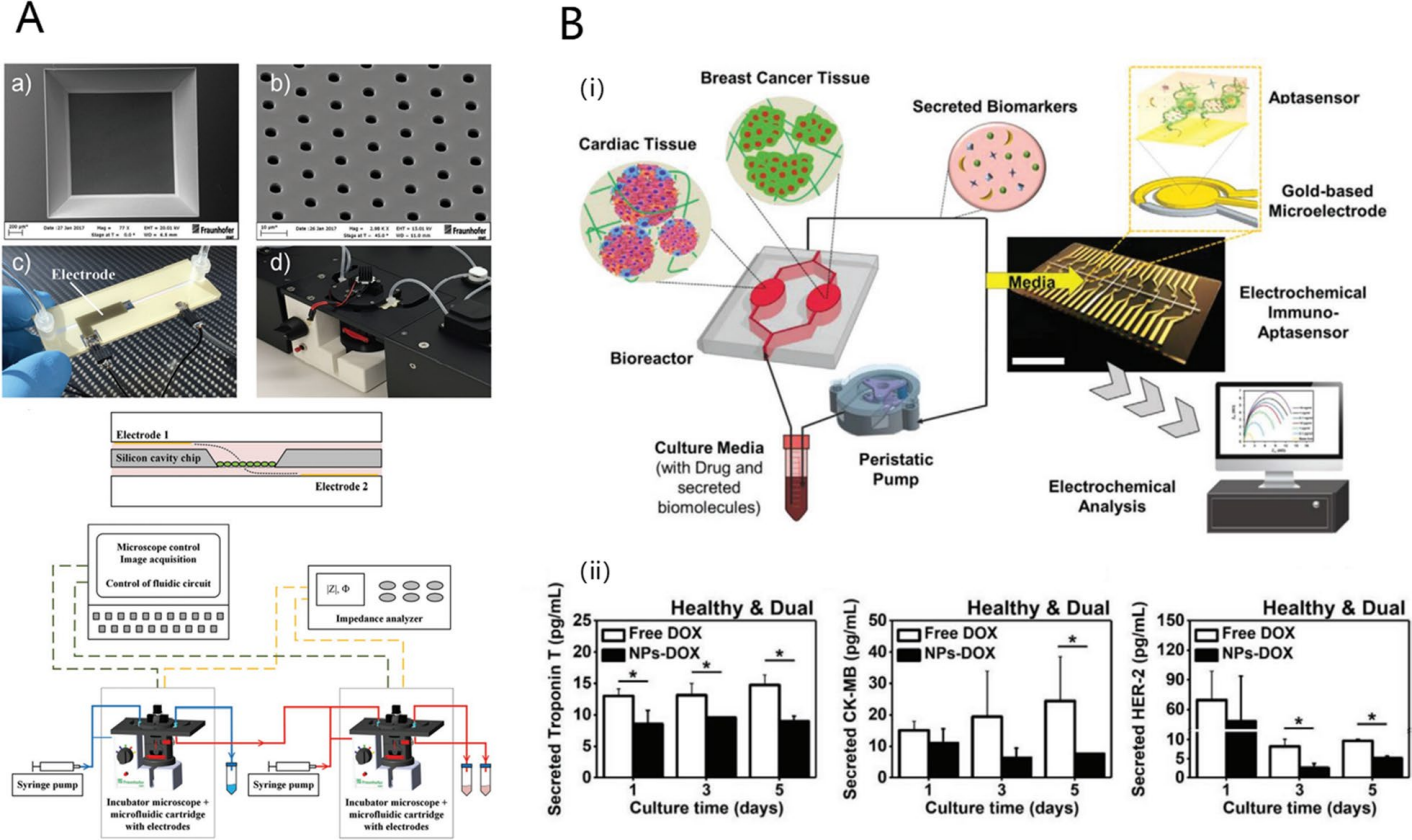
Drug safety evaluation in TOCs. **A** Setup of the microfluidic cartridge and miniaturized incubator microscope platform. Reproduced with permission [60], Copyright 2021, John Wiley and Sons. **B** Heart-cancer-on-a-chip platform to characterize CIC, (i) schematic diagram of design principles, (ii) EC measurement of biomarkers after treatment with DOX. Reproduced with permission [61], Copyright 2021, John Wiley and Sons

Drug safety is an important index in the drug development process, especially for antitumor drugs. Animal models have been used for predicting drug safety. However, poor preclinical to clinical translation is inevitable owing to interspecific variation [102]. There is a growing need for animal-free and high-throughput approaches for safety assessment. Implanting human-derived cells in liver-on-a-chip platforms has proven to be species-specific and potential in predicting multiple types of human hepatoxicities [103]. Microfluidic TOC platforms can also monitor toxicity non-invasively and in real-time by integrating electronic sensors. Kohl et al. developed a microfluidic platform for in vitro cell culture composed of a silicon chip with integrated electrodes and microcavities (Fig. 6A) [60]. The results obtained with three human cell lines, A549 (lung), HepG2 (liver), and TH-1 (kidney), showed the platform is suitable for the label-free assessment of cytotoxic effects. Miniature microscopes within each module could monitor cell morphology and proliferation. The electrodes integrated in the microfluidic channels allow for non-invasive monitoring of barrier integrity in real time. Each microfluidic cell culture module can be operated individually or connected to each other in a flexible manner. The interconnection of different modules was designed to mimic systemic exposure, providing an alternative to animal testing in risk assessment studies. Another study focused on the cardiotoxicity caused by doxorubicin (DOX) [61]. Cardiotoxicity is one of the most serious side effects of chemotherapy in breast cancer (BC). Current methods for monitoring chemotherapy-induced cardiotoxicity (CIC), as well as model systems for CIC platforms established in vivo or in vitro, fail to detect early signs of CIC. Lee et al. presented a heart-cancer-on-a-chip platform integrating induced pluripotent stem cell (iPSC)-derived healthy or fibrotic cardiac tissues with BC tissues using a microfluidic-based channel, with electrochemical (EC) immuno-aptasensors to monitor tissue responses to chemotherapeutic drugs in a non-invasive manner (Fig. 6B). A series of specific biomarkers for myocardial injuries, such as Troponin T and CK-MB, was monitored by EC immuno-aptasensors to characterize DOX-induced CIC. Compared with conventional ELISA method, the detection limits of EC sensors were lower and sensitivities were higher, indicating significant advantages in early-stage CIC prediction. Further, the platform was treated with DOX-loaded NPs to verify its multifunctionality. The NPs showed reduced CIC compared to free DOX with less production of Troponin T and CK-MB. Given the outstanding accuracy of the platform, it may enable early detection and prediction of CIC in individual patients in the future.

### Drug resistance

One of the critical, but difficult challenges in the clinical treatment of cancer is the heterogeneous responses and drug resistances of tumor cells to chemotherapeutic drugs [104]. A number of factors can induce drug resistance, such as hypoxia, drug P-gp efflux and tumor-associated stromal cells [105–107]. TOCs are promising for reproducing these conditions in vitro. For example, Baek et al. evaluated the effect of the anti-hypoxic microparticles in a 3D patient-derived glioblastoma spheroid-loaded microchannel network chip (GBM-chip) [62]. They first concluded a new mechanistic insight into the effects of hypoxia on epigenetic alterations and consequent progressive drug resistance by the GBM-chip. The hypoxic condition for different sizes of spheroids on 3D static culture chips were compared and the hypoxia inside the spheroid was clearly observed when the size reached 500 μm. The expression of O-6-methylguanine-DNA methyltransferase (MGMT), the primary protein that induces alkylating drug resistance, was highly promoted with severe hypoxia condition of spheroids in that size. The brain microvascular circulation in vivo may supply enough oxygen, thereby decreasing hypoxia within GBM tissues. This effect could not be verified by 2D static culture, while GBM-chip could recapitulate in vivo perfusion environments by equipping the tumor spheroid wells with an interconnected network of microchannels. These results verified that hypoxia could be partially alleviated in the perfusion culture; however, the extent was not sufficient. Further, the oxygen-releasing microparticles were loaded to the GBM-chip and suppressed hypoxia and MGMT expression in the chip. Consequently, resistance to a high concentration of temozolomide (TMZ) differed between treatments with or without oxygen microparticle loading. In another study, Agarwal et al. developed a 3D vascularized tumor model capable of mimicking the vascular and stromal microenvironment of tumors in vivo to study the effect of vascularization and stromal cells on drug resistance [63]. The drug resistance levels of the TOC to free DOX were 4.7 and 139.5 times higher than those of avascular microtumors and 2D cultured cancer cells, respectively, indicating the effect of stromal cells and 3D culture on drug resistance in tumor models. The effect of NP delivery on drug resistance was further evaluated in the 3D vascular tumor model. Compared with free DOX, the drug resistance for DOX-NPs was 16-fold lower, indicating that the destructive effect of DOX-NPs on 3D vascular tumors was significantly better than that of free DOX. These results validated the importance of changes in the TME for evaluating NP delivery and drug resistance. Similarly, Shin et al. fabricated a tumor-microenvironment-on-chip (T-MOC) with microfluidic channels to mimic vascular, lymphatic vessels and middle tumor interstitium [64]. The drug response and resistance of DOX-HCl and DOX-loaded HA-targeted NPs were investigated in this T-MOC and a 2D monolayer model. Compared with that in 2D monolayer culture, the survival rate of cells cultured on the T-MOC platform was higher, irrespective of the use of free DOX or NPs. Three different breast cancer cells (i.e., MCF-7, MDA-MB-231, and SUM-159PT) implanted into the T-MOC showed differences in drug responses for the same DOX concentration and these differences were not captured by the 2D model. The cell-type specific drug response and resistance indicated that the T-MOC may be able to present some features of the TME related to drug resistance, representing an advance over 2D model.

## Current issues related to tumor-on-a-chip platform

In the past few decades, nanotechnology-based NDDS for cancer therapy have been rapidly developed. However, the clinical translation of these nanomedicine is still limited by the poor correlation between preclinical in vitro and animal evaluation results and clinical in vivo responses. To address the demand for accurate screening models for nanomedicines, TOC platforms have gained great attention in recent years. To further support the potential of this novel technology for practical applications in clinic trials, the following issues should be resolved in future studies.

The aim of TOC is to mimic real tumor tissue in vivo; however, commercial immortal tumor cell lines, which lack the heterogeneity of patient tumors, are commonly used. To re-create the TME in vitro, other types of cells (e.g., vascular cells, stroma cells, and even immune cells) may be added to the TOC. However, these cells are obtained from different sources, without uniform standards. In recent years, patient-derived organoids have raised the possibility of personalized medicine. They can preserve the genetic, proteomic, and morphological features of the original tumors. However, there are still many technical challenges in tumor organoid culture, such as in vitro expansion and the strict conditions for the long-term culture of neoplastic cells [108, 109]. It is necessary to address these limitations for the meaningful application of TOC to evaluate novel nanomedicines.

The small cell mass of TOC limits the accuracy and sensitivity of conventional analytical methods, such as chromatography quantitation or western blotting. Optical assays are commonly used to evaluate NP responses in TOC; however, the results are qualitative and do not provide insight into specific molecular mechanisms. A key challenge and a new research direction is to combine TOC with advanced sensing components. Integrated models with electrochemical sensors or other types of sensors could provide a basis for non-invasive real-time assays. Using these technologies, nanomedicine responses or toxicity in TOC yield an in-depth understanding at the molecular level.

In addition, it is still challenging to obtain good correlations between in vitro results gained from TOC and clinical in vivo responses. This can be attributed to that human body involves the cooperation of multi-organs, while TOCs are always implemented alone in nanomedicine evaluations. Quantitative parameters such as drug pharmacokinetics (PKs), pharmacodynamics (PDs) or minimal effective doses, could not be reliably predicted without essential physiological processes. Integrating multi-organ-chips might provide reliable information with the aid of computational modelling [50]. In addition, for computational modelling to be comparable with clinical results, close cooperation between chip engineers, clinical doctors, and pharmaceutical companies is needed.

In the development of TOCs, some issues related to chip fabrication need to be addressed. Most chips are fabricated using PDMS. Although this material has multiple advantages, its main drawback is the high absorption to hydrophobic molecules [110, 111]. Most chemotherapeutic drugs are small hydrophobic molecules. Although drug loading into NPs is expected to prevent the direct contact of drugs with the chip, questions still remain. For example, in a comparison of the effects of free drugs and drug-loaded NPs, the role of PDMS absorption is unclear. Further studies are needed to develop advanced materials as alternatives [112].

Although great progress has been made in TOC research, it is still worth to think deeply which is more important of high-throughput or high-content manners. To make the TOC more biomimetic, chip systems are getting increasingly complex, resulting in difficulties with respect to operation and reproducibility. This can be antithetical to the original intention of the TOCs. Hence, it is important to consider the balance between the recapitulation of the TME and engineering complexity in further TOC design. Furthermore, the standards and criteria for each chip are not uniform. Generally, they can only be compared by themselves without horizontal comparison. The final aim of TOC design is to achieve clinical translation, and this requires standardized methods that can be scaled up.

## Conclusion

Although NDDS for anticancer drugs have advanced significantly in the past few decades, most of these platforms have failed in clinical trials owing to insufficient antitumor effects or safety problems. To resolve these critical issues and provide effective alternative models for preclinical studies, tumor-on-a-chip platforms have emerged. As highlighted in this review, the combination of TOC and anticancer nanomedicines offers an accurate and reliable approach for promising preclinical nanomedicines, not only for the prediction of overall therapeutic efficacy but also for evaluations of every step of drug delivery. With increasing research inputs in the field from governmental organizations and pharmaceutical giants, TOCs are expected to play essential roles in nanomedicine development.
